# Reconstruction of the gastric passage by a side-to-side gastrogastrostomy after failed vertical-banded gastroplasty: a case report

**DOI:** 10.1186/1752-1947-2-185

**Published:** 2008-06-02

**Authors:** Christopher Soll, Markus K Müller, Stefan Wildi, Pierre-Alain Clavien, Markus Weber

**Affiliations:** 1 Department of Visceral and Transplantation Surgery, University Hospital Zurich, Raemistrasse, CH-8091 Zürich, Switzerland

## Abstract

**Introduction:**

Vertical-banded gastroplasty, a technique that is commonly performed in the treatment of morbid obesity, represents a nonadjustable restrictive procedure which reduces the volume of the upper stomach by a vertical stapler line. In addition, a textile or silicone band restricts food passage through the stomach.

**Case presentation:**

A 71-year-old woman presented with a severe gastric stenosis 11 years after vertical gastroplasty. We describe a side-to-side gastrogastrostomy as a safe surgical procedure to restore the physiological gastric passage after failed vertical-banded gastroplasty.

**Conclusion:**

Occasionally, restrictive procedures for morbid obesity cannot be converted into an alternative bariatric procedure to maintain weight control. This report demonstrates that a side-to-side gastrogastrostomy is a feasible and safe procedure.

## Introduction

Vertical-banded gastroplasty (VBG) is a commonly performed surgical technique that has been used for many years to treat morbid obesity [1]. It represents a nonadjustable restrictive procedure, which reduces the volume of the upper stomach using a vertical stapler line. In addition, a textile or silicone band restricts the passage of food through the stomach. VBG is usually performed by an open approach and it is not adjustable. Owing to these facts it has been almost completely replaced by the laparoscopic adjustable gastric banding (LAGB) technique in recent years [2].

Here we report the case of a 71-year old woman who presented 11 years after VBG with an inability to swallow solid food. A gastrographin swallow revealed a dilated distal oesophagus and a lack of oesophagogastric passage. The patient was treated surgically with a side-to-side gastrogastrostomy to re-establish the physiological gastric passage. This method demonstrates a simple and safe technique avoiding extensive reconstructive surgery.

## Case presentation

A 71-year-old woman was admitted to our clinic with recurrent postprandial emesis, heartburn for 3 months and inability to swallow solid food. In addition, she had lost 12 kg in this time. Her body weight at admission was 72 kg. Past medical history revealed a VBG for morbid obesity in 1995. A month earlier she had been treated medically for aspiration pneumonia.

**Figure 1:**
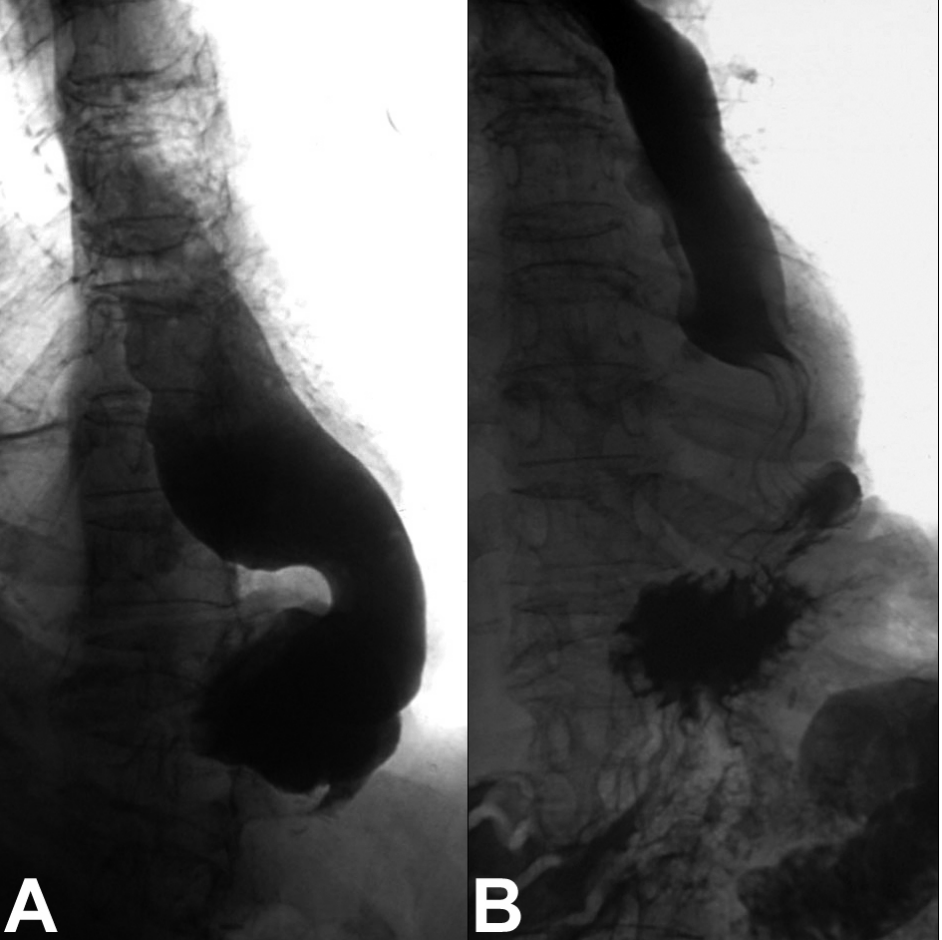
Gastrographin swallow before and 3 months after the operation. (A) Extensive dilatation of the oesophagus with a small infradiaphragmatic pouch. (B) Normal food passage through the distal oesophageal sphincter and a normal sized oesophagus.

A gastrographin swallow showed an extensive dilatation of the oesophagus with a small infradiaphragmatic pouch (Figure 1). The contrast did not pass below the diaphragm and stopped at the level of the oesophageal sphincter, mimicking a pseudoachalasia. Abdominal and thoracic computed tomography confirmed the diagnosis of oesophageal dilatation with a stenosis at the level of the VBG. Two gastroscopic pneumodilatations were performed without success and therefore she was referred for surgical revision.

**Figure 2:**
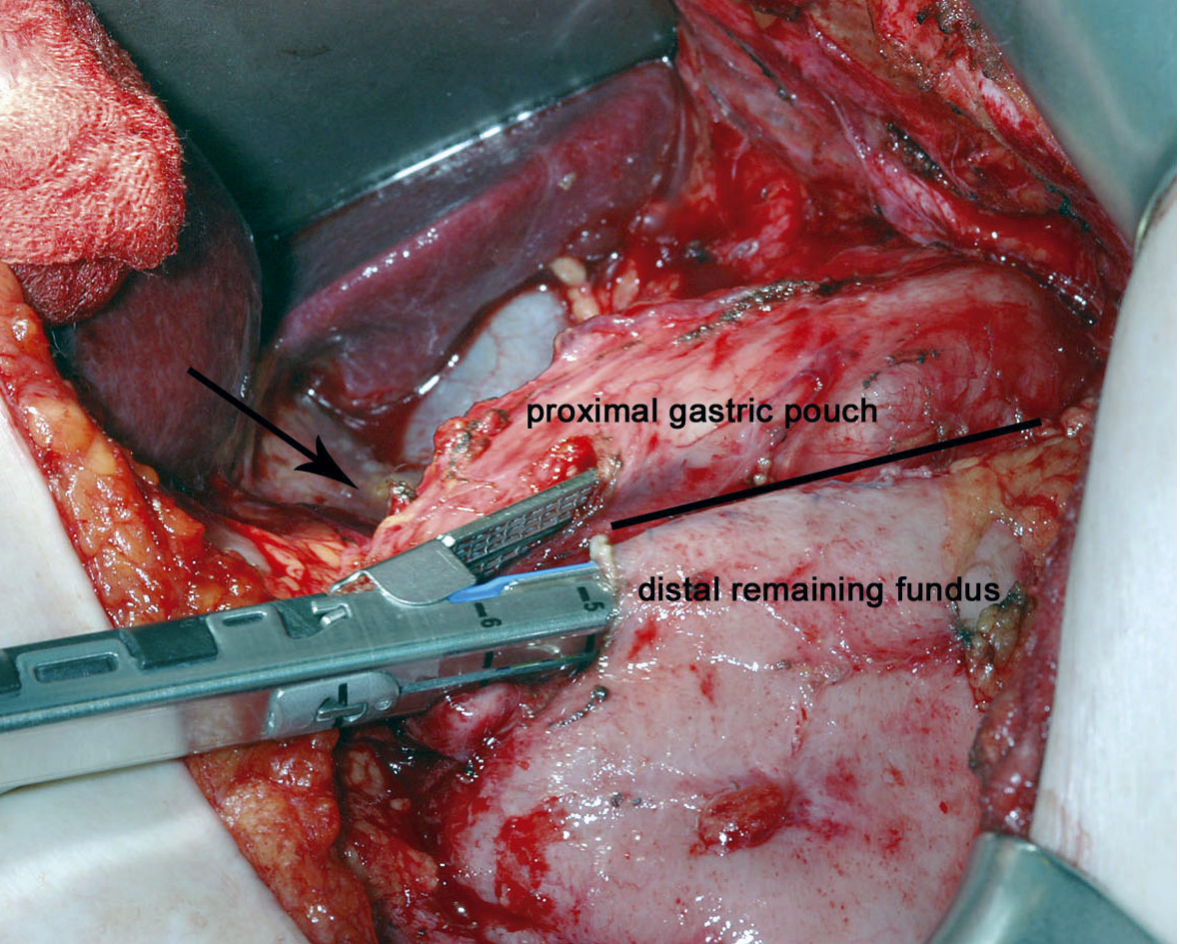
Side-to-side gastrogastrostomy with a 60 mm stapler line. The arrow indicates the position of the textile band occluding the gastric passage. The black bar marks the original stapler line.

The gastric band was identified through an upper midline laparotomy. Simple removal of the textile band would not have re-established the gastric passage sufficiently because of extensive scar tissue. In addition, there was a high risk of gastro-oesophageal perforation due to massive adhesions. Therefore, a side-to-side anastomosis of the proximal gastric pouch with the remaining fundus of the stomach was performed using a linear stapler. The anastomosis was created on the anterior wall of the stomach, leaving the original staple line and the band untouched. We used a 4-0 absorbable running suture to close the incisions for the introduction of the stapler (Figure 2).

The postoperative course was uneventful. She was able to swallow solid food without any of the pre-existing symptoms. Her body weight increased to 77 kg. A gastrographin swallow 3 months after the operation demonstrated a normal gastrointestinal passage (Figure 1).

## Discussion

The VBG was first established by Mason in 1982 [3] and represents a nonadjustable restrictive procedure which reduces the volume of the upper stomach by a vertical stapler line. In addition, a textile or silicone band restricts food passage through the stomach. Until the introduction of LAGB in the early 1990s, this technique was a commonly used surgical procedure among restrictive therapies for morbid obesity [1]. Complications after VBG include leakage, infections, vertical staple-line disruption, pouch dilatation, band erosion and gastric stenosis. Infection and erosion should be treated by band removal. Conversion from VBG to LAGB has been described in severe cases of stenosis or band erosion. Band removal after vertical staple line disruption and pouch dilatation may lead to an increase in weight and, similar to the management of failed LAGB, a conversion to a Roux-en-Y gastric bypass (RYGB) may be indicated in order to reduce weight [2,4]. A narrow outlet or complete gastric stenosis occurs in up to 20% of all patients after VBG [5].

The patient reported here developed a complete gastric stenosis 11 years after VBG. In order to re-establish the ability to swallow solid food and improve her quality of life, an anastomosis between the pouch and the remnant stomach was performed. This procedure was chosen because of the age of the patient, and also because she refused a conversion to an RYGB. Thus, the gastric passage was restored, avoiding time-consuming resection of staple lines and band materials as well as complex reconstructive surgery. The vascularisation of the stomach facilitates good conditions for healing of an anastomosis.

## Conclusion

Occasionally, restrictive procedures for morbid obesity cannot be converted into an alternative bariatric procedure to maintain weight control, either because patients refuse a conversion or because of the age of the patient and other reasons. This report demonstrates that a side-to-side gastrogastrostomy is a feasible and safe procedure to effectively restore the physiological gastric passage after failed VBG.

## Abbreviations

LAGB: laparoscopic adjustable gastric banding; RYGB: Roux-en-Y gastric bypass; VBG: vertical-banded gastroplasty.

## Competing interests

The authors declare that they have no competing interests.

## Consent

Written informed consent was obtained from the patient for publication of this case report and accompanying images. A copy of the written consent is available for review by the Editor-in-Chief of this journal.

## Authors' contributions

CS outlined and wrote the manuscript, MKM and MW treated the patient, performed the operation and contributed to the critical review of the paper, SW was involved in drafting the manuscript and critical revision, PAC gave final approval of the version to be published. All authors read and approved the final manuscript.
